# Impact of Ocrelizumab on Disease Progression, Memory Improvement, and Quality of Life in Patients with Relapsing-Remitting Multiple Sclerosis: A Longitudinal MRI and Clinical Criteria Analysis

**DOI:** 10.3390/diseases12060127

**Published:** 2024-06-16

**Authors:** Amanda Claudia Schuldesz, Ram Kiram Maganti, Raluca Tudor, Amalia Cornea, Mihaela Prodan, Ana-Olivia Toma, Roxana Manuela Fericean, Mihaela Simu

**Affiliations:** 1 Doctoral School, “Victor Babes” University of Medicine and Pharmacy Timisoara, 300041 Timisoara, Romania; amanda.schuldesz@umft.ro (A.C.S.); mihaela.prodan@umft.ro (M.P.); 2 School of General Medicine, Sri Devaraj Urs Academy of Higher Education and Research, Kolar 563101, India; ramkiran.maganti11@gmail.com; 3 Discipline of Neurology, “Victor Babes” University of Medicine and Pharmacy Timisoara, 300041 Timisoara, Romania; tudor.raluca@umft.ro (R.T.); amalia.cornea@umft.ro (A.C.); simu.mihaela@umft.ro (M.S.); 4 Discipline of Dermatology, “Victor Babes” University of Medicine and Pharmacy Timisoara, 300041 Timisoara, Romania; 5 Department of Dermatology, Timisoara Municipal Emergency Hospital, 300254 Timisoara, Romania; manuela.fericean@umft.ro

**Keywords:** multiple sclerosis, monoclonal antibody, quality of life, neurology

## Abstract

Multiple sclerosis (MS) is a chronic, progressive neurological disorder that significantly impacts quality of life and functionality. Ocrelizumab, a monoclonal antibody targeting CD20-positive B cells, has emerged as a treatment for relapsing-remitting MS (RRMS). This study aimed to assess the impact of ocrelizumab on disease progression and quality of life over a longitudinal course, utilizing clinical criteria and magnetic resonance imaging (MRI) analyses. Conducted at the Neurology Department of Pius Brinzeu Clinical Emergency Hospital in Western Romania from 2020 to 2023, this observational study enrolled 93 patients with RRMS who commenced ocrelizumab therapy. The study employed the Expanded Disability Status Scale (EDSS) and MRI to evaluate disease progression, while quality of life was assessed using the World Health Organisation Quality of Life (WHOQOL) questionnaire, Beck Depression Index (BDI), and MOCA scales. Significant improvements were observed post-treatment. EDSS scores decreased from 4.61 to 4.08 (*p* = 0.038), indicating reduced disability. MRI analyses showed a substantial decrease in expansive lesions (from 67.74% to 26.88%, *p* < 0.001) and an increase in stationary lesions (from 32.26% to 73.12%, *p* < 0.001). Quality of life improvements were notable in the physical (from 58.42 to 64.84, *p* = 0.005) and environmental domains (from 63.21 to 68.44, *p* = 0.033). Cognitive functions, assessed via Montreal Cognitive Assessment (MOCA), showed a significant total score increase from 20.38 to 22.30 (*p* < 0.001). Subgroup analysis revealed more pronounced effects in females and younger patients, with a significant reduction in depressive symptoms measured by BDI scores (from 14.35 to 11.62, *p* = 0.003). Ocrelizumab significantly reduced disease activity and disability in RRMS patients, as demonstrated by improvements in EDSS scores and MRI findings. Quality of life and cognitive functions also showed considerable enhancements. These findings support ocrelizumab’s efficacy in not only managing MS symptoms but also improving overall patient well-being.

## 1. Introduction

Multiple sclerosis (MS) is a chronic autoimmune disorder that affects the central nervous system, leading to a wide array of neurological symptoms and significant morbidity [1,2]. Disease-modifying therapies (DMTs) have evolved as a cornerstone in managing MS, with ocrelizumab being a relatively recent addition approved for treating both relapsing-remitting MS (RRMS) and primary progressive MS (PPMS) [3,4]. Ocrelizumab, a humanized monoclonal antibody targeting CD20-positive B cells, has shown promise in reducing disease activity as observed through clinical trials [5,6].

The real-world effectiveness and impact of ocrelizumab on long-term outcomes in MS patients remain areas of active research [7,8]. Studies leveraging real-world data have started to fill these gaps, providing insights into the drug’s performance outside the controlled environment of clinical trials [9,10]. Real-world studies have generally supported the controlled trial data, suggesting that ocrelizumab can reduce relapse rates and magnetic resonance imaging (MRI) shows evidence of disease activity in a broader, less-selected patient population [11,12].

Despite these advances, the effects of ocrelizumab on patient-reported outcomes, particularly quality of life (QoL), are less well understood. Quality of life in MS patients is influenced by various factors including physical disability, cognitive function, and emotional well-being [13,14]. Previous studies have indicated that improvements in clinical and radiological measures of disease activity do not always correlate strongly with QoL improvements [15,16]. Moreover, the application of advanced MRI techniques in clinical practice has enhanced the understanding of MS pathophysiology and the monitoring of disease progression. Longitudinal MRI assessments provide a quantifiable measure of disease activity and progression, which are essential for evaluating the effectiveness of treatments such as ocrelizumab [17,18].

Therefore, the objective of this study is to analyze the impact of ocrelizumab on disease progression in MS patients over a longitudinal course, utilizing a combination of clinical criteria and MRI analyses. Another aim of this study is to determine the impact of treatment on the quality of life of the analyzed patients.

## 2. Materials and Methods

### 2.1. Research Framework and Ethical Considerations

This longitudinal observational study was conducted at the Neurology Department of the “Pius Brinzeu” Clinical Emergency Hospital in Western Romania, focusing on patients diagnosed with multiple sclerosis who have been treated with ocrelizumab (Ocrevus^®^, Genentech, Inc., which is based in South San Francisco, CA, USA). The study spanned from 2020 to 2023, with a goal to evaluate the impact of ocrelizumab on disease progression and quality of life as determined by clinical and MRI assessments. Ethical approval for this study was obtained from the hospital’s Institutional Review Board, in compliance with the ethical guidelines outlined in the Declaration of Helsinki.

Data were collected retrospectively from both paper and digital medical records of patients who had initiated treatment with ocrelizumab during the specified period. Inclusion in the study required that patients had comprehensive pre- and post-treatment MRI data available. Prior to data collection, informed consent was obtained from all participants, permitting the use of their personal and medical records for research purposes.

The current study adhered to the following PICO statement: Population: Adults diagnosed with multiple sclerosis. Intervention: Treatment with ocrelizumab. Comparison: Pre-treatment baselines of disease activity and quality of life measurements. Outcome: Changes in disease progression (as measured by Expanded Disability Status Scale (EDSS) scores and MRI findings) and quality of life (as assessed through validated quality-of-life scales).

### 2.2. Participant Selection and Definitions

This longitudinal panel study exclusively involved participants who received ocrelizumab treatment. There was no comparison group receiving either no treatment or an alternative therapeutic intervention. Inclusion criteria for this study were defined as follows: (1) confirmed diagnosis of multiple sclerosis based on the 2017 McDonald criteria [19]; (2) initiation of ocrelizumab treatment within the study period from 2020 to 2023; (3) availability of complete medical and MRI records before and after the initiation of ocrelizumab treatment; (4) Patients with relapsing-remitting disease.

Exclusion criteria included: (1) patients with other demyelinating diseases or neurodegenerative disorders that could confound the assessment of ocrelizumab’s effects; (2) previous participation in any clinical trials for other experimental MS treatments within 6 months prior to the start of ocrelizumab treatment; (3) missing baseline or follow-up MRI data; (4) patients who discontinued ocrelizumab treatment due to reasons unrelated to disease progression or side effects (e.g., personal preference, relocation); (5) incomplete medical records or lack of informed consent; (6) primary-progressive disease.

### 2.3. Study Variables

The study captured data across several variables to assess the effectiveness and impact of Ocrelizumab treatment in MS patients. Demographic variables included age at the onset of treatment and gender of the participants. Clinical variables comprised the commencement of treatment, prior treatments for MS, and detailed timelines for the initiation and the most recent follow-up of Ocrelizumab therapy.

Disease progression was measured using the EDSS [20] scores recorded at the initiation of therapy and during the latest follow-up, providing insights into the progression or stabilization of the disease. MRI findings served as another essential variable, evaluating changes in brain morphology over the course of treatment to monitor disease activity.

Safety and tolerability were assessed by tracking adverse effects, including their types and severities, and whether the treatment was continued or discontinued, along with the reasons for any discontinuation. Immunological monitoring involved tracking changes in lymphocyte counts over time. Additionally, the JC virus status was monitored to assess the risk of progressive multifocal leukoencephalopathy. Quality of life was evaluated using validated scales, measuring various aspects such as physical, mental, and emotional health, as well as cognitive function.

### 2.4. Study Surveys

To assess the quality of life in MS patients treated with Ocrelizumab, we utilized three validated instruments, each focusing on different domains of well-being and cognitive function. The World Health Organization Quality of Life (WHOQOL) scale is comprehensive, encompassing four broad domains: physical health, psychological health, social relationships, and environment [21]. Each domain consists of several items scored on a Likert scale. Scores are then transformed on a scale from 0 to 100, where higher scores denote a better QoL. This scale provides a nuanced view of the patient’s perceived quality of life across multiple aspects of their daily living.

The Beck Depression Inventory (BDI) specifically targets the psychological domain, assessing the presence and severity of depressive symptoms [22]. It includes 21 items, each describing a specific symptom or attitude. Responses are scored on a scale from 0 to 3, with higher scores indicating more severe depressive symptoms. The total score can range from 0 to 63, with established thresholds categorizing the results as minimal, mild, moderate, or severe depression. This tool is critical for identifying depression, which can affect treatment outcomes and overall well-being.

The Montreal Cognitive Assessment (MOCA) is designed to quickly assess cognitive functions that are commonly impacted in MS, including memory, attention, language, executive functions, visuospatial abilities, and orientation [23]. The MOCA includes a variety of tasks, each contributing to a composite score out of 30 points. A score of 26 or above is generally considered normal, while lower scores indicate some level of cognitive impairment.

### 2.5. Statistical Analysis

The statistical analyses conducted in this study involve comparing measures on the same individuals at two distinct time points. Statistical analyses were performed using SPSS version 26.0 (IBM Corp., Armonk, NY, USA). Continuous variables, such as EDSS scores, lymphocyte counts, and WHOQOL scores, were described using means and standard deviations (SD). Categorical variables, including gender distribution and previous treatment types, were presented as frequencies and percentages. To assess changes in continuous outcomes from treatment onset to one year, paired *t*-tests were applied. Changes in categorical variables were evaluated using the Chi-square test or Fisher’s exact test to compare proportions between baseline and follow-up. Subgroup analyses were conducted to explore the differential effects of ocrelizumab across various demographic and clinical subpopulations, employing stratified analysis techniques. A *p*-value of less than 0.05 was considered statistically significant for all tests.

## 3. Results

The study enrolled 93 patients with relapsing-remitting multiple sclerosis, exhibiting a mean age of 37.8 years with a standard deviation of 9.23 years, and an age range of 23 to 55 years. The gender distribution within the cohort revealed a higher proportion of females (61.29%, n = 57) compared to males (38.71%, n = 36). The average age at MS diagnosis was 29.3 years, with a standard deviation of 7.45 years. The mean disease duration for the cohort was 8.2 years, with a standard deviation of 4.56 years.

Regarding prior exposure to the JC virus, which is relevant due to its association with progressive multifocal leukoencephalopathy in MS treatments, 22.58% of the participants (n = 21) had a history of JC virus infection. The self-reported side effects of Ocrelizumab were documented in 18.27% of the participants (n = 17). The analysis of previous treatments revealed diverse prior MS management strategies, with Interferon beta-1a being the most commonly reported previous treatment (26.88%, n = 25), followed closely by Natalizumab (25.81%, n = 24), and Teriflunomide (23.66%, n = 22). Glatiramer acetate was used by 21.51% of the patients (n = 20). Less commonly used treatments included Dimethyl fumarate (4.30%, n = 4), and even fewer patients had been treated with newer or less common therapies such as Daclizumab, Ozanimod, Fingolimod, and Cladribine, each used by approximately 1% of the cohort, as presented in Table 1.

The mean EDSS scores, which measure the severity of disability, decreased significantly from 4.61 (SD ± 1.80) at treatment onset to 4.08 (SD ± 1.66) after one year (*p* = 0.038). MRI disease features showed remarkable changes, with the proportion of patients having stationary lesions increasing from 32.26% at baseline to 73.12% at one year, while those with expansive lesions decreased from 67.74% to 26.88% (*p* < 0.001). Clinically, there was a notable improvement in patient conditions. The percentage of patients classified as stable increased from 18.28% at the start of treatment to 72.04% after one year, while those experiencing a relapse decreased from 60.22% to 13.98%. In this context, disease relapse was considered a new or worsening neurological symptom in multiple sclerosis that lasts for at least 24 hours and occurs in the absence of fever or infection. Additionally, patients with disease progression, as defined by an EDSS increase, dropped from 21.51% to 13.98% (*p* < 0.001). The average lymphocyte count showed a significant decrease from 3472.5 (SD ± 278.6) per mm3 at treatment initiation to 2613.6 (SD ± 287.4) per mm3 after one year (*p* < 0.001), as presented in Table 2.

The data showed significant improvements in certain quality-of-life domains over the one-year period. The physical domain of WHOQOL scores, which assesses the physical health and levels of physical pain and discomfort, showed an increase from an average of 58.42 (SD ± 14.79) at treatment onset to 64.84 (SD ± 16.05) after one year, with a statistically significant *p*-value of 0.005. The environmental domain also exhibited a significant improvement, with scores rising from 63.21 (SD ± 15.83) at the beginning of the treatment to 68.44 (SD ± 17.34) after one year (*p* = 0.033).

**Figure 1:**
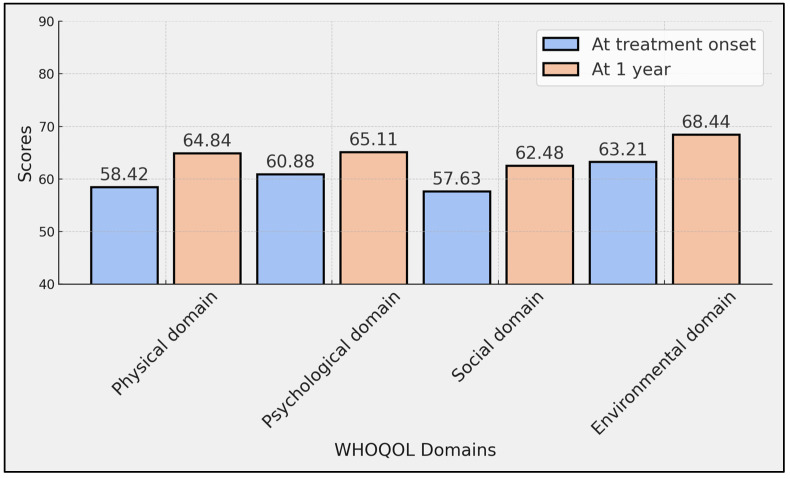
Comparison of WHOQOL scores at treatment onset with ocrelizumab and at 1 year.

Conversely, improvements in the psychological and social domains, while positive, did not reach statistical significance. The psychological domain, which assesses mental health and emotional well-being, increased from 60.88 (SD ± 16.29) to 65.11 (SD ± 17.16) with a *p*-value of 0.086. The social domain, related to personal relationships and social support, rose from 57.63 (SD ± 22.91) to 62.48 (SD ± 24.85) with a *p*-value of 0.168 (Table 3, Figure 1).

The BDI total score, which assesses depressive symptoms, significantly decreased from 14.35 at the onset of treatment to 11.62 after one year, as indicated by a *p*-value of 0.003. Significant improvements were also observed in several MOCA sub-scores over the course of the study. Specifically, execution scores increased from 2.76 to 2.98 (*p* = 0.003), naming scores from 2.47 to 2.79 (*p* < 0.001), language scores from 1.69 to 1.97 (*p* = 0.011), abstraction scores from 1.23 to 1.41 (*p* = 0.007), recall scores from 2.34 to 2.76 (*p* < 0.001), and orientation scores from 5.68 to 5.91 (*p* = 0.008).

**Figure 2:**
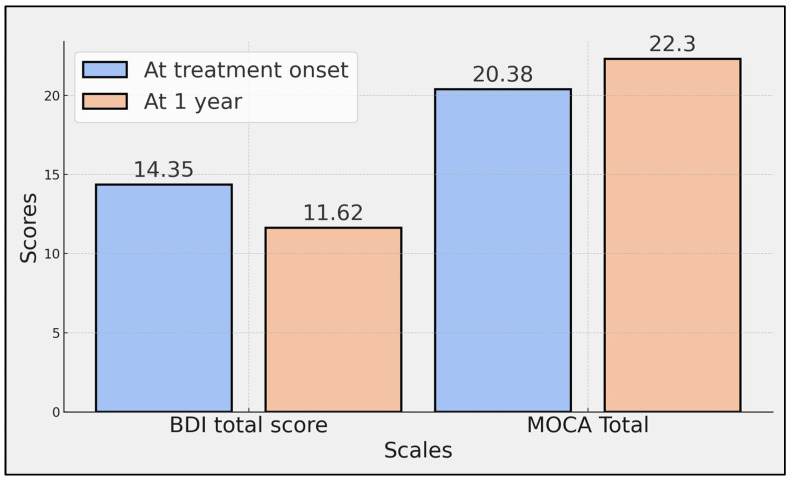
Comparison of total BDI and MOCA scores at treatment onset with ocrelizumab and at 1 year.

However, the scores in the attention component, which improved from 4.21 to 4.48, did not reach statistical significance (*p* = 0.051). The total MOCA score, representing the aggregate cognitive performance, significantly increased from 20.38 at treatment onset to 22.30 at one year (*p* < 0.001), indicating a comprehensive improvement in overall cognitive function among the patients (Table 4, Figure 2).

The study found significant gender differences in the reduction of the EDSS scores, a measure of disability severity in MS. Males experienced an average EDSS score reduction of −0.48, while females saw a more substantial reduction of −0.75, with a significant *p*-value of 0.037. Patients diagnosed at age 25 or younger showed more pronounced improvements in both cognitive function and quality of life compared to those diagnosed after age 25. Specifically, the younger subgroup improved by an average of +2.5 points on the MOCA score (*p* = 0.045) and +6.1 points on the WHOQOL scores (*p* = 0.086), whereas the older subgroup saw improvements of +1.8 and +4.2 points, respectively.

The effect of previous treatment regimens was also significant, with patients previously treated with natalizumab showing a −29.6% reduction in new MRI-detected lesions compared to a −11.3% reduction in those treated with first-line therapies (*p* = 0.015). The analysis of multivariate interactions revealed that females treated with natalizumab benefited the most in terms of quality-of-life improvements, showing a +6.9 point increase in WHOQOL scores compared to a +3.2 point increase in other subgroups (*p* = 0.032), adjusted in a full model including all confounders. Similarly, patients diagnosed with MS at an earlier age demonstrated a more substantial cognitive improvement, with a +3.3 point increase on the MOCA scale compared to a +1.4 point increase in those diagnosed later (*p* = 0.018), also adjusted for all confounders (Table 5).

## 4. Discussion

### 4.1. Literature Findings

The findings from this study highlight the impact of ocrelizumab on multiple sclerosis disease progression and quality of life, revealing significant reductions in EDSS scores and MRI lesion activity over a one-year treatment period. The reduction in EDSS scores, from an average of 4.61 at treatment onset to 4.08 after one year, illustrates a modest but statistically significant improvement in disability. This aligns with the primary pharmacological targets of ocrelizumab, aimed at reducing inflammatory activity in the central nervous system. Furthermore, the shift in MRI disease features, with a marked increase in stationary lesions and decrease in expansive lesions, suggests a robust response to ocrelizumab in stabilizing disease activity, a critical factor in managing MS progression.

In terms of quality of life, the differential impacts observed across WHOQOL domains also provide valuable insights. The significant improvements in the physical and environmental domains contrast with the non-significant changes in the psychological and social domains. This could suggest that while ocrelizumab effectively addresses physical health and aspects of the living environment, the complexities of psychological well-being and social interactions may require additional support or longer treatment duration to manifest noticeable improvements. These findings stress the importance of a holistic approach in MS treatment, where pharmacological interventions are complemented by psychological and social support services to optimize overall patient outcomes.

The subgroup analysis further enriches our understanding of the nuanced effects of ocrelizumab, revealing that certain demographics, such as gender and age at diagnosis, play a significant role in treatment efficacy. The more pronounced EDSS score reduction in females compared to males, and the greater cognitive and quality of life improvements in younger patients, underscore the variability in therapeutic response, which could be influenced by factors such as hormonal differences, disease duration, and baseline disability. These results suggest that personalized MS management strategies, considering patient-specific characteristics, could enhance treatment outcomes.

Lastly, the analysis of previous treatments and multivariate interactions reveals that prior use of natalizumab and the synergistic effects of gender and previous treatments significantly influence the outcome of ocrelizumab therapy. Patients previously treated with natalizumab showed better MRI outcomes, potentially indicating a carry-over effect or a synergistic mechanism when switched to ocrelizumab. Additionally, the interaction effects, particularly in women who received natalizumab, leading to higher improvements in quality of life, highlight the potential for targeted therapeutic strategies based on a patient’s treatment history and demographic factors. These findings advocate for the need to consider past treatment exposures and gender-specific responses when planning future therapeutic regimens, potentially guiding more effective, tailored treatment approaches in relapsing-remitting MS.

In another study by Bonnie I. Glanz et al. [24], notable improvements were recorded over a 12-month treatment period. The study involved 130 participants who were assessed using the Medical Outcomes Study SF-36 and Neuro-QoL patient-reported outcome (PRO) measures. Initially, participants showed low HRQOL across various domains, but significant enhancements were observed by the 12-month mark, particularly in the SF-36 Role-Physical, General Health, Vitality, Role-Emotional, Mental Health, and Mental Component Summary. Similarly, in Neuro-QoL, improvements were significant in Positive Affect, Anxiety, Emotional and Behavioral Dyscontrol, and Fatigue.

In a study led by Carrie M. Hersh et al. [25], researchers compared the time to clinically meaningful improvement in Quality of Life in Neurological Disorders (Neuro-QoL) between patients treated with natalizumab and ocrelizumab. The results indicated that patients receiving natalizumab experienced significantly quicker improvements in cognitive function (event time ratio: 0.37 [0.24–0.57], *p* < 0.001), sleep disturbance (0.45 [0.28–0.72], *p* = 0.001), social role participation (0.37 [0.21–0.66], *p* = 0.001), and social role satisfaction (0.5 [0.31–0.8], *p* = 0.004) compared to those on ocrelizumab. Conversely, another study, SYMptom Burden on Ocrelizumab, a Longitudinal Study (SymBOLS), conducted by Ilya Kister and colleagues, explored symptom changes across two consecutive ocrelizumab infusion cycles [26]. They found no significant worsening of symptoms, with NeuroQoL and SymptoMScreen scores remaining stable or slightly improved. This finding challenges the notion of symptom ‘wearing off’ towards the end of the infusion cycle, suggesting that any perceived exacerbation may stem from natural symptom fluctuations or attribution bias rather than a physiological decline.

In a retrospective cross-sectional study conducted by J. Ingwersen et al. [27], the impact of ocrelizumab on progression independent of relapse activity (PIRA) in relapsing-remitting multiple sclerosis was investigated. The study analyzed 97 patients with a mean follow-up of 29 months and found that 23.5% of these patients developed confirmed disability accumulation, with the majority (87%, n = 20) experiencing PIRA as opposed to relapse-associated worsening. Interestingly, shorter disease duration prior to ocrelizumab treatment and a lower number of previous disease-modifying treatments were associated with an increased probability of developing PIRA, suggesting a distinctive progression pathway influenced by early intervention. This study highlighted that PIRA was the main driver of disability accumulation in the cohort, aligning closely with phase 3 trial results, thus supporting the extrapolation of randomized controlled trial outcomes to real-world settings. In a contrasting regional study by Beatriz Garcia-Cañibano et al. [7], the effectiveness and safety of ocrelizumab were evaluated in an Arab population with multiple sclerosis, yielding good efficacy and tolerability. This retrospective observational study included 60 patients with a median follow-up of 19 months, achieving No Evidence of Disease Activity status in 73% of cases by the first year.

In a real-world study conducted by Angel P. Sempere and colleagues in Spain, ocrelizumab’s effectiveness, safety, and tolerability were assessed in patients with PPMS and relapsing multiple sclerosis [28]. The study involved 70 patients and demonstrated high efficacy of ocrelizumab, with 94% of relapsing MS patients achieving no evidence of disease activity status within the first year. Most patients (98.6%) showed an absence of T1 Gd-enhancing lesions at the first 4–6-month MRI follow-up, indicating a significant reduction in inflammatory activity. The treatment was well-tolerated with minor infusion-related reactions and infections being the most common adverse events. In contrast, Stephen L. Hauser et al.’s [29] OPERA I and II trials, which were randomized phase 3 studies comparing ocrelizumab with interferon beta-1a in relapsing MS, showed ocrelizumab’s superior efficacy in reducing the annualized relapse rate by approximately 46% and 47%, respectively, and significantly lowering the risk of confirmed disability progression. The trials also demonstrated a profound reduction in new gadolinium-enhancing lesions, highlighting ocrelizumab’s potent anti-inflammatory effect. These studies collectively affirm ocrelizumab’s robust clinical effectiveness in both controlled trial and real-world settings, underscoring its significant impact on disease activity and progression in MS patients. However, the need for ongoing surveillance of its long-term safety profile was emphasized, given the potential serious adverse effects observed.

### 4.2. Study Limitations

This study, while comprehensive, has several limitations that should be acknowledged. The observational nature of the study limits the ability to definitively establish causality between ocrelizumab treatment and observed outcomes. The sample size, although adequate, restricts the generalizability of the findings to all populations with multiple sclerosis. Additionally, the single-center design may introduce biases linked to specific regional medical practices or demographic characteristics that are not representative of wider populations. The reliance on self-reported quality-of-life measures might also introduce subjective biases that could affect the accuracy of the results. Lastly, the study duration of one year provides limited insight into the long-term effects of ocrelizumab, especially concerning sustained disability progression and quality of life. Future studies should consider multicenter designs to enhance the generalizability of findings, include larger sample sizes to improve statistical power, and incorporate a control group receiving standard MS treatments for better causality assessment. Additionally, extending the follow-up period and integrating psychological and social support could offer deeper insights into the long-term impacts of ocrelizumab. Complementing self-reported data with objective assessments would also help minimize bias and provide more accurate results.

## 5. Conclusions

The findings from this study are supportive of the effectiveness of ocrelizumab in reducing disease activity and improving quality of life among patients with relapsing-remitting multiple sclerosis. Statistically significant improvements in disability scores, MRI lesion activity, and cognitive functions highlight ocrelizumab’s potential as a transformative treatment for MS. However, future research should aim to include larger, multicenter trials with longer follow-up periods to better understand the long-term benefits and potential risks of ocrelizumab. Additionally, exploring the differential effects of ocrelizumab across various MS subtypes and identifying predictive markers of response to treatment could optimize patient-specific therapeutic strategies, enhancing personalized medicine approaches in multiple sclerosis care.

## Figures and Tables

**Table 1:** Characteristics of patients with relapsing-remitting multiple sclerosis.

Variables	n = 93	SD/%
Age (mean ± SD)	37.8	9.23
Age range	23–55	–
Gender		
Male	36	38.71%
Female	57	61.29%
Age at MS diagnosis (mean ± SD)	29.3	7.45
Disease duration (mean ± SD)	8.2	4.56
Previous JC virus infection	21	22.58%
Ocrelizumab side effects *	17	18.27%
**Previous treatment**		
Glatiramer acetate (Copaxone)	20	21.51%
Interferon beta-1a (Rebif/Avonex/Betaferon)	25	26.88%
Teriflunomide (Aubagio)	22	23.66%
Natalizumab (Tysabri)	24	25.81%
Daclizumab	2	2.15%
Dimethyl fumarate (Tecfidera)	4	4.30%
Ozanimod	1	1.08%
Fingolimod (Gilenya)	1	1.08%
Cladribine (Mavenclad)	1	1.08%

SD—Standard Deviation; MS—Multiple Sclerosis; *—Ocrelizumab side effects were described as infusion reactions, skin infections, and other infections.

**Table 2:** Comparison of disease features at treatment onset with ocrelizumab and at 1 year.

Variables	At Treatment Onset (n = 93)	At 1 Year (n = 93)	*p*-Value
EDSS (mean ± SD)	4.61 ± 1.80	4.08 ± 1.66	0.038
**MRI disease features**			<0.001
1 (stationary lesions)	30 (32.26 %)	68 (73.12%)	
2 (expansive lesions) *	63 (67.74%)	25 (26.88%)	
**Clinical criteria**			<0.001
1 (stable)	17 (18.28%)	67 (72.04%)	
2 (after acute episode)	56 (60.22%)	13 (13.98%)	
3 (progression)	20 (21.51%)	13 (13.98%)	
Lymphocytes (10^3^/mm^3^)	3472.5 ± 278.6	2613.6 ± 287.4	<0.001

EDSS—Expanded Disability Status Scale; MRI—Magnetic Resonance Imaging; SD—Standard Deviation; *—Expansive lesions were defined as lesions that are increasing in size or number, indicating active disease progression.

**Table 3:** Comparison of WHOQOL scores at treatment onset with ocrelizumab and at 1 year.

WHOQOL	At Treatment Onset (n = 93)	At 1 Year (n = 93)	*p*-Value
Physical domain	58.42 ± 14.79	64.84 ± 16.05	0.005
Psychological domain	60.88 ± 16.29	65.11 ± 17.16	0.086
Social domain	57.63 ± 22.91	62.48 ± 24.85	0.168
Environmental domain	63.21 ± 15.83	68.44 ± 17.34	0.033

WHOQOL—World Health Organization Quality of Life; SD—Standard Deviation.

**Table 4:** Comparison of BDI and MOCA scores at treatment onset with ocrelizumab and at 1 year.

Scales	At Treatment Onset (n = 93)	At 1 Year (n = 93)	*p*-Value
BDI total score	14.35 ± 6.87	11.62 ± 5.64	0.003
**MOCA**			
Execution	2.76 ± 0.52	2.98 ± 0.49	0.003
Naming	2.47 ± 0.68	2.79 ± 0.57	<0.001
Attention	4.21 ± 1.04	4.48 ± 0.82	0.051
Language	1.69 ± 0.82	1.97 ± 0.63	0.011
Abstraction	1.23 ± 0.46	1.41 ± 0.45	0.007
Recall	2.34 ± 0.92	2.76 ± 0.74	<0.001
Orientation	5.68 ± 0.62	5.91 ± 0.55	0.008
Total	20.38 ± 2.95	22.30 ± 2.54	<0.001

BDI—Beck Depression Inventory; MOCA—Montreal Cognitive Assessment.

**Table 5:** Subgroup analysis.

Variable	Subgroups	Outcome	Change/Effect	*p*-Value	Confounders Adjustment
Gender	Male vs. Female	EDSS score reduction	Males: −0.48, Females: −0.75	0.037	Age, disease duration
Age at MS diagnosis	≤25 years vs. >25 years	MOCA score improvement	≤25: +2.5 points, >25: +1.8 points	0.045	Gender, previous treatment
		WHOQOL Improvement	Improvement scores: ≤25: +6.1 points, >25: +4.2 points	0.086	Gender, disease duration
Previous treatment	Natalizumab vs. First-line	MRI lesion progression	Reduction in new lesions: Natalizumab users: −29.6%, First-line: −11.3%	0.015	Age, gender
Multivariate interaction	Gender * Natalizumab	WHOQOL improvement	Improvement scores: Females with Natalizumab: +6.9 points, Others: +3.2 points	0.032	Full model (all confounders)
	Age at Diagnosis * MOCA	Cognitive outcomes	Improvement scores: Early-diagnosed: +3.3 points, Late-diagnosed: +1.4 points	0.018	Full model (all confounders)

MRI—Magnetic Resonance Imaging; MOCA—Montreal Cognitive Assessment; MS—Multiple Sclerosis; WHOQOL—World Health Organization Quality of Life; EDSS—Expanded Disability Status Scale.

## Data Availability

Data available on request.
